# Serum cytokines in primary new daily persistent headache and chronic migraine: a case control study

**DOI:** 10.3389/fneur.2025.1547124

**Published:** 2025-04-01

**Authors:** Sanjay Cheema, Edmund Garr, Dwij Mehta, Miles Chapman, Melanie Hart, Michael P. Lunn, Valeria Iodice, Manjit Singh Matharu

**Affiliations:** ^1^Headache and Facial Pain Group, University College London (UCL) Queen Square Institute of Neurology, London, United Kingdom; ^2^Neuroimmunology and CSF Laboratory, University College London Hospitals NHS Trust, London, United Kingdom; ^3^Autonomic Unit, UCL Queen Square Institute of Neurology, London, United Kingdom

**Keywords:** cytokines, biomarkers, neuroinflammation, new daily persistent headache, migraine

## Abstract

**Background:**

New daily persistent headache (NDPH) is a poorly understood headache disorder which often begins after a viral infection and has been hypothesised to have an immune basis. Several studies have found differences in peripheral serum cytokine levels between healthy controls and patients with other primary headache disorders such as migraine. We sought to measure peripheral levels of cytokines and chemokines in NDPH, chronic migraine (CM), and healthy controls, to identify any changes which could be biomarkers of inflammation in NDPH.

**Methods:**

We performed an observational prospective case control study measuring serum cytokine levels in three age and sex matched groups of 40 patients each: patients with NDPH, patients with CM with similar headache frequency and severity, and healthy controls. We accounted for confounders such as infective or autoimmune illness, vaccinations, comorbidities, and medications. We used a 30-PLEX human cytokine immunoassay to measure the concentration of 30 cytokines and chemokines. The primary outcome measures were differences in median concentrations of TNF-*α*, IL-6, and IL-8 between the three groups, as these are the three cytokines found to differ between chronic migraine and healthy controls in a recent meta-analysis.

**Results:**

Age and sex distribution was balanced between the groups. The NDPH and daily CM groups were balanced on headache frequency, severity, and duration. The concentration of IL-6 was higher in both NDPH (median 0.603 pg/mL) and CM (median 0.642 pg/mL), than in healthy controls (median 0.399 pg/mL), but there was no difference between the NDPH and CM groups. There were no significant differences in TNF-*α*, IL-8, or any of the other cytokines tested between the three groups.

**Conclusion:**

Peripheral cytokine levels do not appear to be helpful biomarkers in differentiating NDPH from daily CM, and the lack of differences to healthy controls does not lend support to the hypothesis of neuroinflammation being involved in the pathophysiology of NDPH.

## Introduction

A growing body of evidence has suggested that the immune system may be involved in the pathophysiology of primary headache disorders, in particular the persistence of chronic daily headache (1). It is surmised that this may help explain why patients with a daily headache are often refractory to standard headache treatments.

A recent systematic review and meta-analysis found that patients with migraine have significantly higher peripherally circulating interleukin (IL)-6, tumour necrosis factor (TNF)-*α*, and IL-8 levels than healthy controls, and patients with tension-type headache had higher TNF-α and transforming growth factor (TGF)-*β* than controls (2). However, these findings are limited by the small numbers of patients and variety of different analysis methods used by each study.

New daily persistent headache (NDPH) is a primary headache disorder with an unknown pathophysiology, which often clinically resembles either chronic migraine (CM) or tension-type headache, but is frequently refractory to migraine treatments (3). NDPH is often precipitated by an infectious illness, and may be associated with comorbid immune-mediated disease (4). Since its first descriptions, immune mechanisms have been suggested to be relevant to the pathogenesis of NDPH (5). NDPH is often precipitated by an infectious illness, and may be associated with comorbid immune-mediated disease (4). NDPH has been associated with higher-than-expected Epstein–Barr virus (EBV) positivity and raised cerebrospinal fluid (CSF) TNF-*α* levels in small studies (6, 7).

We hypothesised that if NDPH is a partly immune-mediated disease, then this might be reflected in raised levels of proinflammatory cytokine levels when NDPH was compared to healthy controls and other forms of chronic daily headache. If this were true it might help explain why patients with NDPH are often refractory to standard headache treatments. If reliable inflammatory biomarkers could be identified this has the potential to develop new pathophysiological hypotheses or trial treatments targeted at blocking neuroinflammation.

### Objectives

To compare levels of peripheral serum inflammatory cytokines in patients with NDPH to those with chronic migraine and healthy controls.

## Materials and methods

### Participants

We performed an observational prospective case control study measuring serum cytokine levels in three age and sex matched groups of 40 patients:

- Group A: Patients diagnosed with NDPH as per International Classification of Headache Disorders 3rd Edition (ICHD-3) criteria (8), with phenotype resembling CM with any duration of illness.
- Group B: Patients diagnosed with CM with a daily headache with any duration of illness.
- Group C: Healthy controls with no more than 4 days per month of tension-type headache, and no history of migraine.

Potential participants in Groups A and B (patients with headache disorders) were identified using review of database of patients previously seen in the headache clinics at the National Hospital for Neurology and Neurosurgery. Potential participants in Group C (non-headache controls) were recruited from relatives and friends of the included patients and co-workers and friends of the researchers. All participants in Groups A and B had resistant headaches, with failure to respond to at least three classes of migraine preventive medication. No patients had refractory migraine as per European Headache Federation consensus definition (9) as no patients had received CGRP monoclonal antibody treatment at the time of enrolment or sample acquisition in this study.

Confounders were identified in the history including current infectious or inflammatory illness, comorbidities including autoimmune, inflammatory, or malignant disease, presence of anxiety or depression, and medications so these could be controlled for or excluded.

Participants in all three groups who had an infective illness or vaccination within the previous 2 weeks were excluded from the study. Patients with active autoimmune/inflammatory disease, malignancy, or using immunosuppressive medication were also excluded, as were patients who had used non-steroidal anti-inflammatory drugs within the previous 48 h. Patients with other potential confounders were not excluded but the presence of confounders was noted.

### Study procedures

A screening appointment was conducted with each participant via telephone to ensure they met inclusion/exclusion criteria and provide patients with information about the study.

Patients were invited for single in-person appointment at a time of their convenience. Patients were required to complete a questionnaire which captured information on demographics; body mass index; current headache frequency, severity, and phenotype; recent infective illness or vaccination; comorbid allergic, inflammatory, or autoimmune disease; malignancy; cardiovascular disease; renal disease; mood disorder; current medications and any recent medication changes.

Venepuncture was performed to obtain serum samples from the antecubital fossa. All samples were taken between 10 am-3 pm and blood was centrifuged and serum separated within 2 h of being collected. Sera were immediately frozen at−80°C.

For the detection and quantification of cytokine and chemokine levels, three sets of MSD (Meso Scale Discovery, Rockville, Maryland, USA) assay kits, with a combined total of 30 analytes, were carried out on serum samples in duplicate as per the manufacturer’s instructions. The use of a SULFO-TAG label conjugated to detection antibodies allowed for ultra-sensitive detection of an electrochemiluminescent signal as the detection method and measured multiple analytes in a single well. In addition, the provision of validated controls in all the assays performed ensured accuracy and precision in our measurements. The following analytes were measured: Eotaxin, Eotaxin-3, GM-CSF, IFN-*γ*, IL-1α, IL-1β, IL-2, IL-4, IL-5, IL-6, IL-7, IL-8, IL-8 (HA), IL-10, IL-12/IL-23p40, IL-12p70, IL-13, IL-15, IL-16, IL-17A, IP-10, MCP-1, MCP-4, MDC, MIP-1*α*, MIP-1*β*, TARC, TNF-α, TNF-β, VEGF-A. This panel was chosen due to the exploratory nature of the study, and inclusion of cytokines which have previously been identified as relevant to primary headache disorders. Samples were analysed in batches which each contained a mixture of the three participant groups.

The primary outcome measure was the difference in levels of TNF-*α*, IL-6, IL-8 between the three groups. These three cytokines were chosen as they were the three which were found to differ between people with migraine and healthy controls in the recent meta-analysis (2).

We planned a secondary analysis within the NDPH group, comparing TNF-*α*, IL-6, IL-8 between those who had an infectious precipitant to their headache and those who did not.

### Statistical analysis

As a study reporting raw serum cytokine levels has not been previously performed in NDPH, we performed a sample size calculation based on the difference in TNF-*α* between CM, episodic migraine, and healthy controls in a previous study (10). Using a *p*-value of 0.05 and power of 0.80, the sample size was calculated as only three participants required in each group. However, since 30 cytokines and chemokines were measured and the exploratory nature of the analysis, we elected to include a larger number of participants. The number was based on the number of patients with the disorders of interest under the care of our clinic at the time, the cost of analysing samples, and numbers included in other previous published studies of cytokines in primary headache disorders. The majority of previous studies were of similar or smaller sample size yet shown statistically significant differences between the groups studied. As the groups were balanced in age and sex distribution, no statistical matching or adjustment for confounders was performed between the three groups.

Baseline characteristics were summarised as means with standard deviation or proportions depending on whether the variable was continuous or categorical; and were compared between the groups using one-way analysis of variance (ANOVA) or Chi-squared test as appropriate. For comparison of cytokine levels between the three groups, the distribution of most cytokines was positively skewed and therefore non-parametric analysis was performed. Descriptive results of the cytokine levels are presented as median and interquartile range, two-group comparisons were performed using the Mann–Whitney test, and three-group comparisons were performed using 2-sided Kruskal-Wallis test. Missing data were not imputed. Statistical analysis was performed using IBM SPSS Version 28.

### Ethical approval and consent

All participants gave written informed consent to participate in the study and publication of anonymised results. Research ethics committee approval for the study was obtained from the London – Queen Square Research Ethics Committee, reference: 22/LO/0300.

## Results

### Participants

Forty participants in each of the three pre-defined groups were enrolled in this study between September 2022 and April 2023. Baseline characteristics are summarised in Table 1. Age and sex distribution was similar in all three groups, and duration of chronic daily headache, headache frequency, and severity was similar between the NDPH and CM groups. One patient in the NDPH group had to be excluded before analysis, as she was discovered to have active rheumatoid arthritis and had recently taken a course of corticosteroids. Two healthy controls were excluded as they reported a history of migraine. Ten (26%) healthy controls reported a history of occasional <4 days per month episodic tension-type headache, which is lower than the 1 year prevalence of tension-type headache in the general population which is usually estimated to be around 40% (11), and therefore representative of the general population.

**Table 1:** Baseline characteristics.

	NDPH-CM	CM	Healthy controls	Between groups comparison
N	39	40	38	N/A
Age, mean +/-SD (years)	43.7 +/−12.8	45.9 +/− 15.7	42.1 +/−16.6	*F* = 0.61, *p* < 0.543
Sex (females)	25 (64.1%)	28 (70.0%)	25 (65.8%)	*X*^2^ = 0.329, *p* = 0.849
BMI, mean +/-SD (kg/m^2^)	28.2 +/−6.9	27.5 +/−7.9	23.7 +/−3.3	*F* = 5.36, *p* = 0.006*
Duration of CDH (years)	8 +/− 12.7	10 +/− 11	N/A	*F* = 0.798, *p* = 0.374
Headache severity (0–10 VRS)	5.5 +/− 3	4.8 +/− 3.5	N/A	*F* = 1.326, *p* = 0.253
Headache frequency (days per 28-day period)	28 (0)	28 (0)	0 (1)	N/A
Current headache at time blood was taken	39 (100%)	40 (100%)	0	N/A
Headache characteristics			N/A	N/A
Throbbing quality	17 (44%)	21 (53%)		
Unilateral pain	9 (23%)	17 (43%)		
Nausea and/or vomiting	22 (56%)	33 (83%)		
Photophobia	34 (87%)	32 (80%)		
Phonophobia	27 (69%)	31 (78%)		
Motion sensitivity	27 (69%)	32 (80%)		
Cranial autonomic symptoms	17 (44%)	21 (53%)		
Aura symptoms	14 (36%)	17 (43%)		
Comorbid medication overuse headache	5 (13%)	6 (15%)	N/A	N/A

Data shown are mean +/− standard deviation or median (interquartile range), unless otherwise specified.

^*^On post-hoc testing with Tukey-b test, significant difference was between both NDPH and CM in comparison to the healthy control group.

BMI, body max index; CDH, chronic daily headache; CM, chronic migraine; NDPH-CM, new daily persistent headache with chronic migraine phenotype; VRS, verbal rating scale.

### Cytokine results

**Figure 1:**
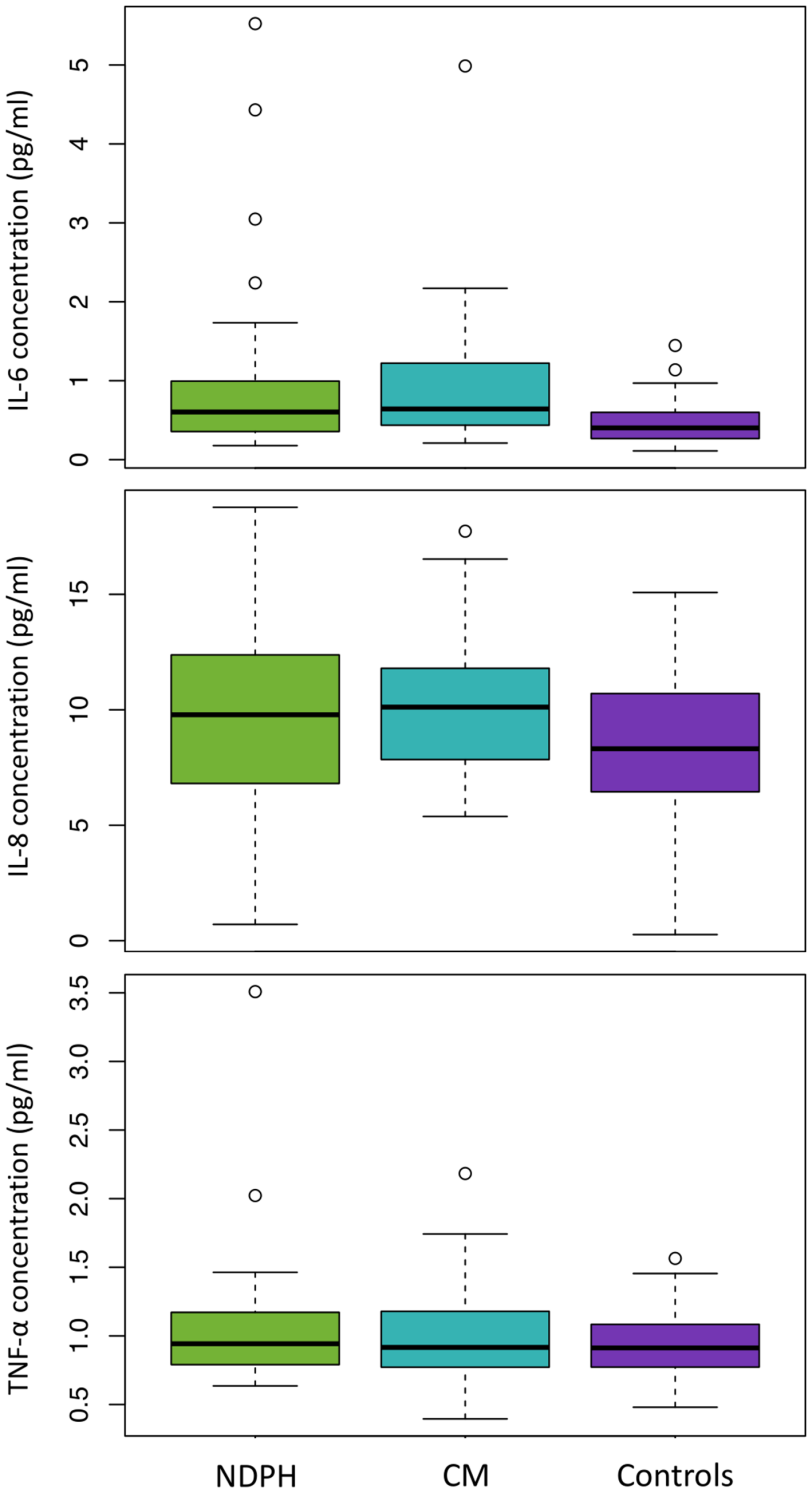
IL-6, IL-8, and TNF-*α* levels in NDPH, CM, and healthy controls. CM, chronic migraine; IL, interleukin; NDPH, new daily persistent headache; TNF, tumour necrosis factor.

IL-6, IL-8, and TNF-*α* were detectable in all 119 participants. The concentration of IL-6 was higher in both NDPH (median 0.603 pg/mL) and CM (median 0.642 pg/mL), than in healthy controls (median 0.399 pg/mL), but there was no difference between the NDPH and CM groups. Concentrations of neither IL-8 or TNF-*α* differed between the three groups (see Table 2; Figure 1).

**Table 2:** Between group comparison of three primary outcome measures.

Biomarker measured	NDPH-CM	CM	Healthy controls (HC)	H statistic	*p* value
IL-6 pg./mL	0.603 (0.690)	0.642 (0.820)	0.399 (0.339)	13.6	0.001 overall 0.007 for HC vs. NDPH <0.001 for HC vs. CM 0.405 for NDPH vs. CM
IL-8 pg./mL	9.78 (5.70)	10.1 (4.22)	8.31 (4.65)	4.51	0.105
TNF-α pg./ml	0.943 (0.423)	0.916 (0.432)	0.901 (0.332)	0.85	0.651

Data are presented as median (IQR) and statistical test used is Kruksal-Wallis test, 2-sided.

An analysis was also conducted for the three primary endpoints with the 10 healthy controls who had low-frequency tension-type headache excluded from the analysis and results were similar.

Several cytokines (IL-12p70, IL-13, IL-1β, IL-2, IL-8(HA), GM-CSF, IL-1α, and IL-5) were undetectable in a large proportion of participants, similarly in all three groups. Concentrations of the other cytokines measured was similar in all three groups (see Table 3).

**Table 3:** Serum concentrations of all 30 cytokines measured.

Cytokine	NDPH	CM	Healthy controls
IFNγ pg/ml	4.86 (7.00)	5.00 (6.46)	4.62 (8.28)
Undetectable	0	0	1
IL-10 pg/mL	0.215 (0.101)	0.216 (0.164)	0.211 (0.128)
Undetectable	0	0	0
IL-12p70 pg/ml	0.004 (0.177)	0.051 (0.143)	0.030 (0.147)
Undetectable	21	16	19
IL-13 pg/mL	0 (0.151)	0 (0.378)	0 (0)
Undetectable	30	29	31
IL-1β pg/ml	0 (0)	0 (0)	0 (0)
Undetectable	30	34	33
IL-2 pg/mL	0.094 (0.190)	0.111 (0.134)	0.048 (0.162)
Undetectable	14	11	15
IL-4 pg/mL	0.026 (0.173)	0.298 (0.014)	0.027 (0.023)
Undetectable	6	1	7
IL-6 pg/mL	0.603 (0.690)	0.642 (0.816)	0.399 (0.339)
Undetectable	0	0	0
IL-8 pg/mL	9.78 (5.70)	10.1 (4.22)	8.31 (4.65)
Undetectable	0	0	0
TNF-α pg/ml	0.943 (0.423)	0.916 (0.432)	0.901 (0.332)
Undetectable	0	0	0
Eotaxin pg/ml	272 (143)	264 (151)	230 (117)
Undetectable	0	0	0
Eotaxin-3 pg/mL	8.51 (5.92)	9.11 (4.07)	8.78 (3.81)
Undetectable	0	0	0
IL-8 (HA) pg/ml	0 (0)	0 (0)	0 (44.5)
Undetectable	34	33	29
IP-10 pg/mL	169 (129)	179 (119)	179 (124)
Undetectable	0	0	0
MCP-1 pg/mL	243 (120)	259 (112)	207 (134)
Undetectable	0	0	0
MCP-4 pg/mL	86.0 (61.0)	102 (62.9)	78.7 (44.1)
Undetectable	0	0	0
MDC pg/ml	987 (426)	1,040 (470)	795 (439)
Undetectable	0	0	0
MIP-1α pg/ml	10.7 (6.13)	11.1 (6.17)	8.81 (4.06)
Undetectable	0	0	0
MIP-1β pg/ml	103 (79.8)	103 (52.5)	113 (70.6)
Undetectable	0	0	0
TARC pg/ml	287 (222)	276 (148)	227 (168)
Undetectable	0	0	0
CM-CSF pg/ml	0 (0.122)	0 (0)	0 (0.078)
Undetectable	24	32	26
IL-12/IL23p40 pg/ml	122 (96.6)	122 (88.6)	102 (60.7)
Undetectable	0	0	0
IL-15 pg/mL	2.17 (0.620)	2.20 (0.659)	2.13 (0.660)
Undetectable	0	0	0
IL-16 pg/mL	259 (149)	253 (150)	219 (137)
Undetectable	0	0	0
IL-17A pg/ml	2.64 (1.90)	2.57 (2.06)	1.98 (1.70)
Undetectable	0	0	0
IL-1α pg/ml	N/A	N/A	N/A
Undetectable	39	40	38
IL-5 pg/mL	0 (0.313)	0 (0.178)	0 (0.153)
Undetectable	21	26	27
IL-7 pg/mL	11.6 (6.68)	10.9 (8.50)	13.1 (7.54)
Undetectable	0	0	0
TNF-β pg/ml	0.205 (0.138)	0.215 (0.222)	0.181 (0.189)
Undetectable	5	6	5
VEGF-A pg/ml	89.4 (94.5)	95.7 (129)	62.3 (67.7)
Undetectable	0	0	0

Values presented as median (IQR) and shown to three significant figures.

Within the NDPH group, nine had an infectious precipitant to their headache. There was no significant difference between those who had an infectious precipitant to those who did not for IL-6 concentration (median 0.65 pg/mL and 0.54 pg/mL, *p* = 0.935), IL-8 concentration (median 9.90 pg/mL and 9.71 pg/mL respectively, *p* = 0.935), or TNF-*α* (median 0.943 pg/mL and 0.930 pg/mL respectively, *p* = 0.831).

## Discussion

The only significant difference in cytokine levels between the three groups included in this study was that IL-6 levels were higher in patients with NDPH and CM than in healthy controls (but no different between NDPH and CM). There were no differences in the other cytokines measured either between the headache groups and healthy controls, or between the NDPH and CM groups. There was also no difference in cytokine levels in patients with NDPH who had a post-infectious onset, although there was only a small number of participants with a post-infectious onset meaning this comparison lacked power. These results altogether suggest that peripherally measured cytokines are not useful biomarkers in differentiating patients with NDPH and CM and do not provide support for the neuroinflammatory hypothesis of NDPH.

The finding of higher IL-6 in both headache groups is consistent with previous studies in migraine (2). A study has shown that calcitonin gene-related peptide (CGRP), a key neuropeptide in migraine pathophysiology highly correlates (*r* = 0.94) with IL-6 levels in patients with migraine (12). However, given that IL-6 levels were similarly raised in both NDPH and CM in this study suggests that it could be a non-specific reaction to pain rather than unique to migraine. IL-6 has also be found to be raised in patients with other chronic pain disorders including fibromyalgia (13). Future studies may benefit from a control group with non-headache pain disorders. Baseline characteristics were similar between the groups with the exception of BMI, which was lower in the control group than both patient groups. Obesity has previously been associated with increased levels of several proinflammatory cytokines including TNF-*α* and IL-6 (14, 15). This could be another factor leading to increased IL-6 in these patient groups.

The only previous study which has measured levels of any cytokine in NDPH measured TNF-*α* in the serum and CSF. Concentrations were not reported but CSF TNF-α was elevated above the normal range in 19 out of 20 patients with NDPH, all of 16 patients with CM, and both of two patients with post-traumatic headache (7), although the reliability of the normative range in the CSF has been questioned (16). TNF-α was above the normal range in the serum in three of 14 NDPH patients and none of five CM patients.

The predominantly negative serum results observed in this study could be indicative of two critical considerations in the pathophysiology of NDPH and CM. Firstly, it is possible that cytokines do not have a significant causal role in these disorders, suggesting that the underlying mechanisms of NDPH and CM may lie beyond the cytokine-mediated inflammatory response. Despite the growing literature on a potential role of inflammatory in migraine, migraine is not considered a classical inflammatory disease and some authors contest the description of “neuroinflammation” as having any role in the pathophysiology of migraine (17).

Secondly, the hypothesis that cytokine activity is primarily localized within the central nervous system, rather than systemically, raises the question of the adequacy of peripheral blood measurements in capturing the true neuroinflammatory processes at play in these headache disorders. Consequently, this underscores the potential utility of cerebrospinal fluid analysis for a more direct investigation of neuroinflammatory markers, which may not be detectable through peripheral blood sampling.

The NDPH and CM patient populations included in this study, who were recruited from a tertiary headache clinic, were resistant to treatment and had a long duration of NDPH prior to samples being taken. It would be interesting to investigate inflammatory or other biomarkers soon after the onset of headache to identify whether markers of inflammation at this point in the disease course have the capability to predict persistence of headache.

The lack of differences in our study between the headache groups and healthy controls contrasts with the results of several other studies with similar or smaller sample sizes, which have shown significant differences in various peripheral serum cytokine levels between patients with CM or CTTH and non-headache controls (see Table 4).

**Table 4:** Previous studies of serum cytokine levels in chronic daily headache syndromes.

First author Year	Markers studied	Study group (*n*)	Control group (*n*)	Main findings
Kocer et al., 2009 (20)	IL-6	CM (66)	HC (45)	IL-6 levels were higher in CM (mean of 67.1 pg/mL in those on topiramate and 44.1 pg/mL in those not on topiramate) compared to normal controls (8.6 pg/mL).
Kocer et al., 2010 (21)	IL-6	CTTH (22) ETTH (20)	HC (37)	IL-6 levels were higher in CTTH (mean 64.8 pg/mL) than ETTH (mean 35.2 pg/mL), and both higher than normal controls (8.6 pg/mL).
Della Vedova et al., 2013 (22)	IFN-y, IL-1*β*, IL-2, IL-4, IL-5, IL-6, IL-8, IL-10, IL-12p70, IL-18, TNF-α and TNF-β	CTTH (56)	HC (42)	There were higher levels of IL-1β in CTTH patients compared to normal controls, but there were no differences in any of the other cytokines measured.
Martami et al., 2018 (23)	TNF-α, CRP	CM (23) EM (20)	HC (40)	Median TNF-a levels were 1.90 pg./mL in those with CM, 0.95 pg/mL in EM, and 0.24 pg/mL in controls (*p* < 0.001). There were no significant differences in CRP.
Han, 2019 (12)	IL-1β, IL-2, IL-6, IL-10, and TNF-α CGRP	Migraine (31 CM, 16 EM)	HC (38)	IL-1β, IL-6, TNF-α, and CGRP were significantly higher in the migraine group than the control group (*p* < 0.05). CGRP levels were strongly correlated with IL-6 levels. There was no significant difference in IL-2 or IL-10 levels. Unfortunately, there was no analysis for differences between those with chronic and episodic migraine.
Togha et al., 2020 (10)	TNF-α, IL-6, CRP	CM (27) EM (44)	HC (19)	The CM group had significantly raised serum concentrations of IL6 (mean = 435.28 pg/mL), CRP (mean = 718.11 pg/mL) and TNF-α (mean = 651.04 pg/mL) than the EM suffers (mean = 340.93, 573.59 and 513.14 pg/mL, respectively) and controls (mean = 259.00, 514.21 and 448.95 pg/mL, respectively) (*p* ≤ 0.001).

CM, chronic migraine; CTTH, chronic tension-type headache; EM, episodic migraine; ETTH, episodic tension-type headache; HC, healthy controls; NDPH, new daily persistent headache; M, migraine (frequency not specified).

The discordance of the negative results in this study with the positive results in the literature could potentially be due to publication bias, although there was no obvious evidence of this in a meta-analysis comparing migraine (not-specifically CM) with healthy controls (2). There may also be methodological explanations. The accurate and sensitive measurement of cytokines has only been possible in the past decade meaning that earlier results are less reliable. MSD cytokine assays have been found to be sensitive and reliable when compared to other assays (18, 19). Various different assays were used in previous studies, and as far as we can interpret from previous publications, no other study has used the same MSD assay as was used in this study.

There is a wide disparity in cytokine concentrations reported in previous studies, which in some cases differ by orders of magnitude (see Table 4). There are several potential explanations for this. Pre-analytics can affect results – some cytokines are hugely unstable and deteriorate if serum is not separated and frozen promptly after collection. The assays (including antibodies, platforms, hardware consumables such as ELISA plates and detection methods) used for analysis of cytokines also vary enormously. Many assay kits are calibrated only to in-house reference standards rather than international standards – e.g. according to the product insert for the MSD assay used in this study, the ratio of MSD concentration relative to the World Health Organisation international standard is 0.106. In some cases, samples have to be diluted in an assay to ensure all the cytokines in the kit are within range, and results may not have been calculated by the dilution factor if one was provided by that assay kit. For these reasons cytokine concentrations are not comparable between studies which used different assays.

A limitation of all cytokine studies is that cytokines can be affected by a myriad of other factors including comorbidities, medications, and how the samples are taken, stored, and analysed. We attempted to account for as many of these factors as possible and with the exception of BMI the groups were otherwise similar at baseline. Samples of the three groups were taken concurrently and analysed in batches with a mix of each group; samples were taken at a similar time of day and centrifuged and analysed at a similar time interval in each of the three groups; patients with active or recently active autoimmune or infectious illness or vaccinations were excluded; patients on immunomodulatory or other medication known to affect cytokine levels were excluded; NDPH and CM patients were recruited from the same clinic population; and analysis of the samples was performed blinded as to the group membership. None of the studies shown in Table 4 accounted for all of these factors, and it is possible that some of the results could be due to unknown confounders. The number of factors which can affect cytokine levels means that they are unlikely to be helpful as diagnostic biomarkers.

## Conclusion

This study shows that peripheral serum cytokines are not useful biomarkers of NDPH, and despite methodological limitations associated with the measurement of peripheral cytokines, do not lend support to the hypothesis of primary NDPH as a disorder involving neuroinflammation.

## Data Availability

The raw data supporting the conclusions of this article will be made available by the authors, without undue reservation.
